# Allostatic load and incident heart failure in the Reasons for Geographic and Racial Differences in Stroke (REGARDS) study

**DOI:** 10.1186/s12872-023-03371-z

**Published:** 2023-07-04

**Authors:** Christine Park, Joanna B. Ringel, Laura C. Pinheiro, Alanna A. Morris, Madeline Sterling, Lauren Balkan, Samprit Banerjee, Emily B. Levitan, Monika M. Safford, Parag Goyal

**Affiliations:** 1 Department of Medicine, New York Presbyterian-Weill Cornell, New York, NY USA; 2 Division of General Internal Medicine, Department of Medicine, Weill Cornell Medicine, New York, NY USA; 3 Department of Medicine, Emory University School of Medicine, Atlanta, GA USA; 4 Department of Cardiology, Beth Israel Deaconess Medical Center, Boston, MA USA; 5 Division of Biostatistics and Epidemiology, Department of Public Health, Weill Medical College of Cornell University, New York, NY USA; 6 Department of Epidemiology, University of Alabama at Birmingham, Birmingham, AL USA; 7 Division of Cardiology, Department of Medicine, Weill Cornell Medicine, 420 E. 70Th St, LH-365, New York, NY 10021 USA

**Keywords:** Allostatic load, Heart failure, Outcomes

## Abstract

**Background:**

Allostatic load (AL) is the physiologic “wear and tear” on the body from stress. Yet, despite stress being implicated in the development heart failure (HF), it is unknown whether AL is associated with incident HF events.

**Methods:**

We examined 16,765 participants without HF at baseline from the REasons for Geographic and Racial Differences in Stroke (REGARDS) cohort. The main exposure was AL score quartile. AL was determined according to 11 physiologic parameters, whereby each parameter was assigned points (0–3) based on quartiles within the sample, and points were summed to create a total AL score ranging from 0–33. The outcome was incident HF event. We examined the association between AL quartile (Q1-Q4) and incident HF events using Cox proportional hazards models, adjusted for demographics, socioeconomic factors, and lifestyle.

**Results:**

The mean age was 64 ± 9.6 years, 61.5% were women, and 38.7% were Black participants. Over a median follow up of 11.4 years, we observed 750 incident HF events (635 HF hospitalizations and 115 HF deaths). Compared to the lowest AL quartile (Q1), the fully adjusted hazards of an incident HF event increased in a graded fashion: Q2 HR 1.49 95% CI 1.12–1.98; Q3 HR 2.47 95% CI 1.89–3.23; Q4 HR 4.28 95% CI 3.28–5.59. The HRs for incident HF event in the fully adjusted model that also adjusted for CAD were attenuated, but remained significant and increased in a similar, graded fashion by AL quartile. There was a significant age interaction (p-for-interaction < 0.001), whereby the associations were observed across each age stratum, but the HRs were highest among those aged < 65 years.

**Conclusion:**

AL was associated with incident HF events, suggesting that AL could be an important risk factor and potential target for future interventions to prevent HF.

**Supplementary Information:**

The online version contains supplementary material available at 10.1186/s12872-023-03371-z.

## Clinical implications

### What is new?

- Increasing levels of allostatic load are incrementally associated with incident heart failure events and is most pronounced in adults younger than 65 years.

### What are the clinical implications?

- Allostatic load, a marker of cumulative stress burden, should be considered as a risk factor for heart failure
- The impact of environmental and societal stressors on incident HF merit further investigation.
- Developing interventions to mitigate AL may be warranted as a strategy to prevent HF

## Introduction

The prevalence of heart failure (HF) continues to rise in the United States, with a predicted increase of 46% from 2012 to 2030, which would result in a HF prevalence that exceeds 8 million by 2030 [1]. This has major implications on morbidity and mortality, as well as cost. Indeed, every year, > 1 million people are hospitalized with HF and 300,000 people die from HF; and it is anticipated that the costs of HF will exceed $69.8 billion by 2030 [1].These data support an urgent need to identify novel targets and strategies for preventing HF.

Social determinants of health (SDOH) has emerged as an important factor influencing the epidemiology of HF [2–4]. Recent work has shown that an increasing number of SDOH are associated with elevated risk for incident HF hospitalization [5, 6]. This observation is hypothesized to result from elevated stress hormones and inflammation markers, which are critical elements in the pathogenesis of HF [6]. Indeed, a potential mechanism underlying the association between SDOH and incident HF is allostatic load (AL). AL is the cumulative physiologic “wear and tear” on the body that results from adapting to chronic stress and is associated with increased mortality in several conditions including cancer and diabetes [7–10]. Indeed, AL has been implicated as an important contributor to observed race-related disparities, in part due to institutionalized racism which can manifest in physiologic dysfunction [10].

While stress and inflammation, through the activation of neurohormonal systems and cytokines, have previously been implicated in the pathophysiology of both heart failure with reduced ejection fraction (HFrEF) and heart failure with preserved ejection fraction (HFpEF), [11, 12] no studies to our knowledge have examined the association between AL and incident HF events. Accordingly, we sought to determine whether AL is associated with incident HF event by examining participants from the prospective bi-racial Reasons for Geographic and Racial Difference in Stroke (REGARDS) study.

## Methods

### REGARDS cohort

The details of the REGARDS study have been previously described [5, 13, 14]. Briefly, it is a national, prospective longitudinal cohort study in which 30,239 community-dwelling adults aged ≥ 45 years were originally enrolled in 2003–2007, with ongoing longitudinal follow-up. Since the study was originally developed to examine geographic and racial differences in stroke mortality, the cohort included an oversampling of the US region known as the Stroke Buckle (North Carolina, South Carolina, and Georgia) and the Stroke Belt (North Carolina, South Carolina, Georgia, Alabama, Mississippi, Tennessee, Arkansas, and Louisiana). Study recruitment occurred via mailing and telephone contact. At enrollment, participants underwent telephone interviews collecting sociodemographic information and medical history; this was followed by an in-home baseline assessment examination with laboratory tests (vitals, electrocardiograms, blood and urine samples), and pill bottle review for medication history. Age, biologic sex, racial background, education level, annual household income, health insurance coverage, diet/exercise habits, and smoking and alcohol history were self-reported. Blood and urine samples collected at the in-home examination were centrally analyzed at a single laboratory at the University of Vermont, and the electrocardiograms were interpreted at Wake Forest University.

REGARDS participants were contacted every 6 months, during which cardiovascular hospitalizations and deaths were ascertained. At the 6-month follow-up telephone calls, interviewers asked participants if they were hospitalized during the prior 6 months, and inquired about the reason for hospitalization. Medical records for potential cardiovascular-related hospitalizations were subsequently retrieved and reviewed by two clinicians to adjudicate HF events. To identify HF hospitalizations, adjudicators considered symptoms (orthopnea, paroxysmal nocturnal dyspnea, nocturnal cough), physical exam findings (dyspnea, orthopnea, paroxysmal nocturnal dyspnea, edema, rales, jugular venous distension), laboratory values (elevated b-type natriuretic peptide), imaging/echocardiography (cardiomegaly, pulmonary vascular congestion, pleural effusions), and medical treatment (diuretic induced weight loss of at least 4.5 kg in 5 days) [13, 15]. Death was ascertained at the time of the routine 6-month follow-up telephone calls, or via letters from a proxy stating that the participant had died. To maximize the accuracy of death ascertainment, study investigators also searched the Social Security Death Index death master file and the National Death Index. Cause of death was adjudicated based on death certificates, interviews with next of kin, and review of medical records from around the time of hospitalization.

### Study population

For this analysis, we included participants who were free of suspected HF at the time of their REGARDS baseline survey. This HF-free cohort was developed using a medication-based algorithm that has previously shown a negative predictive value of > 95% [15]. We excluded participants with missing data on medications and self-reported atrial fibrillation, and participants with HF hospitalizations between the baseline telephone interview and in-home examination, since this precluded determination of whether the patient had suspected HF at baseline; and excluded participants with missing data for any component of the exposure variable (AL).

### Exposure

The primary exposure was AL. We operationalized AL by creating a score that was calculated based on 11 physiologic parameters obtained during the REGARDS baseline assessment, similar to prior work in REGARDS and the National health and Nutrition Examination Survey (NHANES III) examining AL [9]. We selected parameters from major organ systems implicated in the AL response, which include cardiovascular, metabolic, and immune, inflammatory systems [8, 16, 17]; and specifically chose parameters previously shown to increase with stress. Our chosen 11 parameters were among the most frequently utilized parameters across over 20 studies performed within the NHANES dataset [18]. Selected parameters included heart rate, systolic blood pressure, diastolic blood pressure, waist circumference, serum blood glucose level, total cholesterol, high density lipoprotein (HDL) cholesterol, serum albumin, urinary albumin/creatinine ratio, cystatin C, and c-reactive protein. We chose to calculate our AL score by partitioning the values by quartiles, which is the most common method utilized in prior literature. For all parameters except HDL and albumin, numeric values were arranged from lowest to highest, then partitioned into quartiles from the lowest quartile to the highest quartile. For HDL and albumin, since lower values indicated “higher risk”, numeric values were arranged from highest to lowest, then partitioned into quartiles whereby the highest values were assigned to the lowest quartile. We then ascribed points for each parameter based on quartile: the lowest quartiles (Q1) received 0 points, Q2 received 1 point, Q3 received 2 points, and the highest quartiles (Q4) received 3 points assigned. Points from the 11 physiologic parameters were then summed to generate an AL score (minimum: 0; maximum: 33). AL score was then partitioned into quartiles. We chose to assign the parameters by quartiles so that the values would serve as a reflection of our population via normalized clinical values, instead of using traditional cut offs for each parameter, which would instead represent pathology in the causal pathway for heart disease. (i.e. systolic blood pressure of 140 mmHg, which is the established diagnostic cut-off for hypertension).

### Outcome

The main outcome was incident HF events through December 31, 2018. Incident HF events included a composite of adjudicated HF hospitalizations and HF-related deaths. HF-related deaths were confirmed via clinician-adjudicated who examined medical records, death certificates, and interviews with next of kin. We also examined HFrEF and HFpEF-based hospitalizations separately based on diagnostic studies quantifying left ventricular ejection fraction at the time of the hospitalization. HFrEF was defined as a left ventricular ejection fraction (EF) ≤ 50%, or a qualitative report stating reduced EF; and HFpEF was defined as EF > 50% or qualitative report of preserved EF. For the purposes of this study, HFmrEF (EF 41–49%) was grouped with HFrEF given shared pathophysiologic features [19]. Given the classification of HFrEF defined as EF ≤ 40%, we performed sensitivity analyses by repeating the main analyses for AL and incident HFrEF defined by EF ≤ 40%.

### Covariates

We adjusted models for age, sex, and racial background, as well as selected covariates based on the Healthy People 2030 conceptual model for SDOH [20], an approach used in prior work using REGARDS [14]. SDOH-related covariates included variables from each of the following domains of the framework: 1) economic stability (annual household income level); 2) education (highest level of education achieved); 3) social and community context (social isolation status, defined by asking the participants if they have not seen friends or family at least once a month or if they do not have someone to care for them if they are ill/disabled); 4) health care access (health insurance coverage). Given potential impact on incident HF [21], we also adjusted lifestyle behaviors including tobacco smoking, alcohol use, physical activity, and adherence to the Dietary Approaches to Stop Hypertension (DASH) diet. Adherence to the DASH diet was calculated based on the Block 98 Food Frequency Questionnaire (which is administered at the baseline REGARDS assessment) [22].

### Statistical analysis

We examined baseline participant characteristics stratified by AL score quartiles. We compared continuous variables across the four quartiles using ANOVA; and compared categorical variables using a chi-square test.

We estimated Cox proportional-hazard regression models to determine the association between AL quartile and incident HF events. In the fully adjusted model, we adjusted for age, racial background, sex, self-reported social support, geographic region of residence, annual household income level, highest education level achieved, health insurance coverage, tobacco smoking, alcohol use, physical activity, and adherence to DASH. Given the potential for differences in the association of AL and incident HF with age and racial background, we examined for interactions by repeating the analyses with an interaction term in the model and using a Wald test to determine statistical significance. For interactions where the *p*-value was < 0.10, we presented stratified findings.

To determine whether the association between AL and incident HF event remained after accounting for the potential mediating effect of coronary artery disease (CAD), we conducted an additional analysis with the addition of baseline history of CAD as a covariate.

We also examined the association of AL with incident HFrEF and incident HFpEF separately using the Lunn-McNeil extension to Cox proportional hazards models [23]. To handle missingness, we used multiple imputation for missing covariates. As a sensitivity analysis, we repeated the analysis using multiple imputation for the exposure and covariates. The largest percentage of missing data for the covariates were DASH diet quartiles (26%), household income (13%), and social isolation status (6%). Missingness for the remaining covariates were < 2%.

To account for the competing risk of non-HF-related mortality, we performed a sensitivity analysis to examine the association between AL score quartile and incident HF by using the Fine and Gray model for competing mortality risk models [24].

## Results

### Participant characteristics

**Fig. 1:**
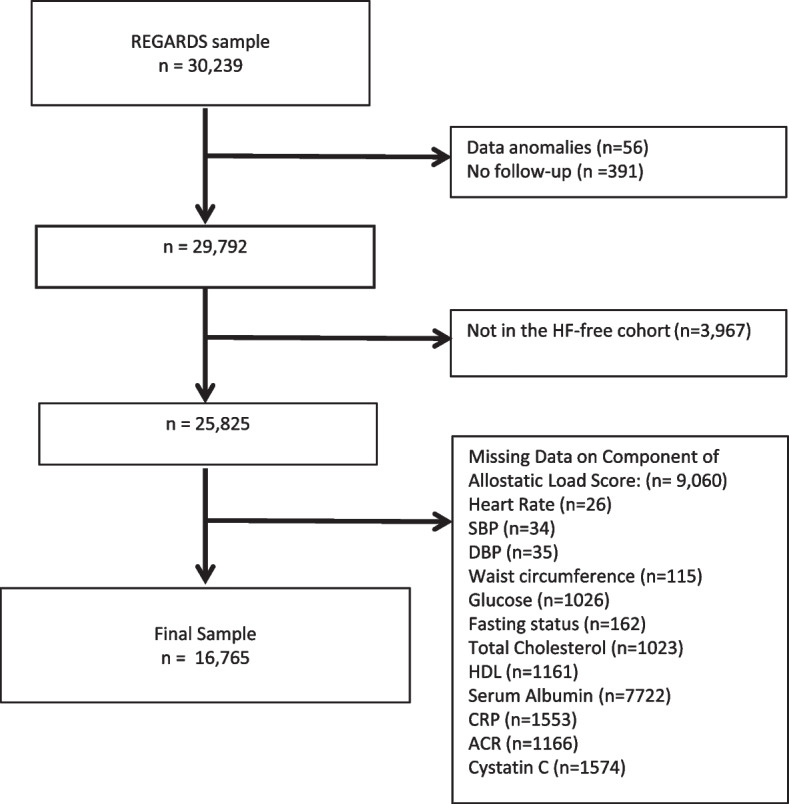
Exclusion cascade. REGARDS participants were excluded if there were data anomalies, no follow-up after study recruitment, had suspected HF (not part of HF-free cohort), or had missing data necessary to calculate the AL score

**Fig. 2:**
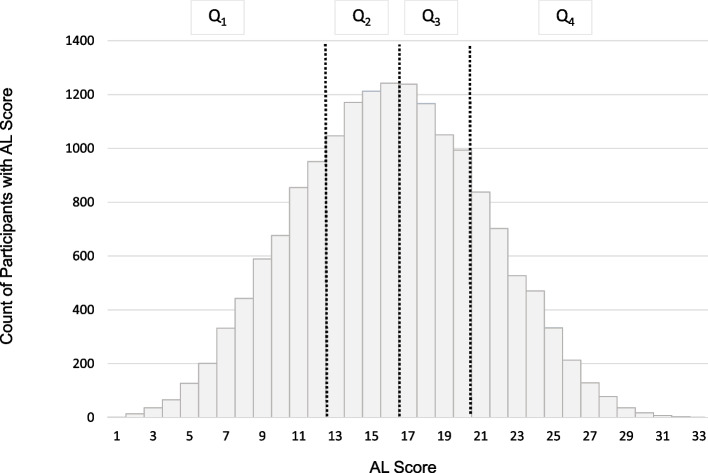
Distribution of AL divided by quartiles (Q1-4) based on frequency within the sample. The frequency follows a normal distribution, with an interquartile range of 12–20, and a median score of 16

Among 30,239 participants from REGARDS, 25,825 were determined to be HF-free at baseline, based on a previously-validated algorithm [15]; among 25,825, 9,060 had missing data on component of the AL score. Accordingly, for this study, we examined 16,765 eligible participants (Fig. 1). The mean age of the participants at baseline was 64 ± 9.6 years, 61.5% were women, and 38.7% were Black participants (Table 1). The median AL score was 16 (interquartile range 12–20). Figure 2 shows the distribution of AL score. Median values of each AL parameter by quartiles are provided in Supplemental Table 1.

**Table 1:** Baseline participant characteristics by allostatic load quartile (Q1, Q4)

Characteristics	All	Q1 (1, 12)	Q2 (13, 16)	Q3 (17, 20)	Q4 (21, 33)	*P*-Value
N	16,765	4290	4672	4449	3354	
Age, mean (SD)	64 (9.6)	61 (9.3)	64 (9.6)	65 (9.7)	64 (9.4)	< 0.001
Female	10,317 (61.5%)	2651 (61.8%)	2792 (59.8%)	2732 (61.4%)	2142 (63.9%)	0.003
Black participants	6492 (38.7%)	1182 (27.6%)	1698 (36.3%)	1905 (42.8%)	1707 (50.9%)	< 0.001
Annual household income						< 0.001
$75,000 and above	3061 (18.3%)	1169 (27.2%)	918 (19.6%)	634 (14.3%)	340 (10.1%)	
$35,000-$74,999	5184 (30.9%)	1438 (33.5%)	1506 (32.2%)	1333 (30.0%)	907 (27.0%)	
$20,000-$34,999	3815 (22.8%)	762 (17.8%)	1025 (21.9%)	1111 (25.0%)	917 (27.3%)	
Less than $20,000	2590 (15.4%)	379 (8.8%)	625 (13.4%)	799 (18.0%)	787 (23.5%)	
Refused	2115 (12.6%)	542 (12.6%)	598 (12.8%)	572 (12.9%)	403 (12.0%)	
Highest level of education achieved						< 0.001
College graduate and above	6184 (36.9%)	2097 (48.9%)	1786 (38.3%)	1390 (31.3%)	911 (27.2%)	
Some college	4607 (27.5%)	1110 (25.9%)	1273 (27.3%)	1241 (27.9%)	983 (29.3%)	
High school graduate	4266 (25.5%)	850 (19.8%)	1187 (25.4%)	1269 (28.5%)	960 (28.7%)	
Less than high school	1698 (10.1%)	232 (5.4%)	423 (9.1%)	547 (12.3%)	496 (14.8%)	
Social isolation	2228 (14.2%)	541 (13.4%)	623 (14.2%)	603 (14.5%)	461 (14.7%)	0.36
No health insurance	1215 (7.3%)	266 (6.2%)	287 (6.2%)	335 (7.5%)	327 (9.8%)	< 0.001
Current smoking	2467 (14.8%)	473 (11.1%)	647 (13.9%)	728 (16.4%)	619 (18.5%)	< 0.001
Alcohol Use						< 0.001
Heavy	686 (4.2%)	230 (5.4%)	190 (4.1%)	158 (3.6%)	108 (3.3%)	
Moderate	5636 (34.2%)	1802 (42.6%)	1663 (36.3%)	1324 (30.4%)	847 (25.7%)	
None	10,153 (61.6%)	2203 (52.0%)	2731 (59.6%)	2876 (66.0%)	2343 (71.0%)	
Times per week of exercise						< 0.001
4 or more per week	4911 (29.7%)	1566 (37.0%)	1407 (30.6%)	1207 (27.5%)	731 (22.1%)	
1 to 3 time per week	6295 (38.1%)	1693 (40.0%)	1814 (39.4%)	1655 (37.7%)	1133 (34.2%)	
None	5328 (32.2%)	973 (23.0%)	1383 (30.0%)	1525 (34.8%)	1447 (43.7%)	
DASH style diet score^a^	24 (4.4)	25 (4.5)	24 (4.3)	24 (4.2)	23 (4.2)	< 0.001

*Abbreviation*: *SD* Standard deviation

^a^Mean and standard deviation

At higher AL quartiles, the proportion of Black participants, income < $20,000, educational attainment of less than high school, current smokers, no exercise per week, and low DASH diet adherence increased in a graded manner (*p* < 0.001) (Table 1). In addition, the highest AL quartile included the highest proportion of women, highest degree of social isolation, and highest proportion of uninsured status. Conversely, the highest AL quartile had the lowest percentage of heavy alcohol use.

### Incident heart failure

**Fig. 3:**
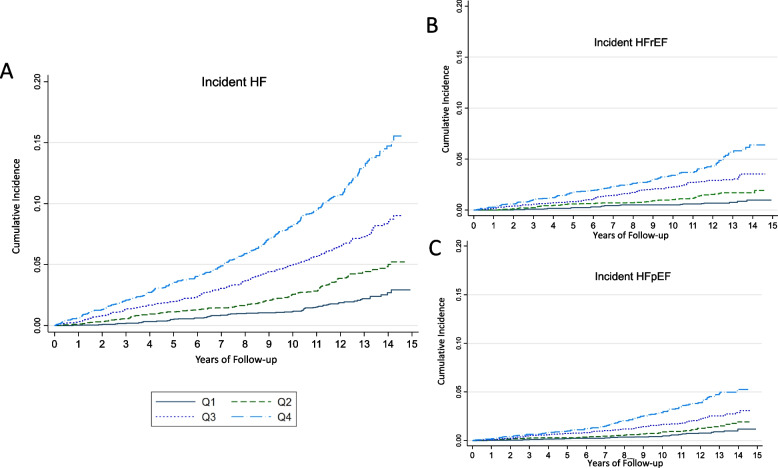
Unadjusted Kaplan–Meier survival curves for incident HF by AL quartile (Q1-Q4). **A** Overall incident HF event, (**B**) Incident HFrEF event, (**C**) Incident HFpEF subtype

Over a median [IQR] follow up of 11.4 [6.9–13.2] years, participants experienced 750 incident HF events including 635 HF-hospitalizations and 115 HF-related deaths. The unadjusted incident rate of HF for AL quartile 1 (Q1) was 1.6 per 1000 person-years, for Q2 was 3.12 per 1000 person-years, for Q3 was 5.6 per 1000 person-years, and for Q4 was 9.6 per 1000 person-years. A Kaplan–Meier curve is shown in Fig. 3. In a fully adjusted model, the hazard ratio (HR) for an incident HF event increased in a graded fashion with higher AL quartiles: Q2 HR 1.49 95% CI 1.12–1.98; Q3 HR 2.47 95% CI 1.89–3.23; Q4 HR 4.28 95% CI 3.28–5.59 (Table 2). Our analysis for an interaction with racial background and AL quartiles was not statistically significant (*p*-value 0.16). Supplemental Table 2 shows models with sequentially adjustment of covariates, demonstrating that the addition of each set of covariates led to mild attenuation with persistence of an association.

**Table 2:** Association between allostatic load and incident HF event stratified by age

		Q1	Q2	Q3	Q4	*P*-Value
		HR (95% CI)	HR (95% CI)	HR (95% CI)	HR (95% CI)	
All	Events/N	72/4,290	148/4,290	239/4,449	291/3,354	
	Rate per 1000 person, years	1.60	3.12	5.60	9.60	
	Unadjusted	Reference	1.97 (1.49, 2.61)	3.58 (2.75, 4.66)	6.25 (4.82, 8.08)	< 0.001
	Fully adjusted	Reference	1.49 (1.12, 1.98)	2.47 (1.89, 3.23)	4.28 (3.28, 5.59)	< 0.001
Age < 65	Events/N	14/2,817	27/2,485	61/ 2,215	107/1,752	
	Rate per 1000 person-years	0.47	1.05	2.78	6.45	
	Unadjusted	Reference	2.23 (1.17, 4.25)	5.99 (3.30, 10.70)	14.12 (8.09, 24.66)	< 0.001
	Fully adjusted	Reference	1.78 (0.93, 3.42)	4.20 (2.33, 7.59)	9.16 (5.17, 16.23)	< 0.001
Age 65—74	Events/N	34/1,068	52/1,448	92/1,443	114/1,071	
	Rate per 1000 person-years	2.98	3.49	6.48	11.96	
	Unadjusted	Reference	1.18 (0.77, 1.82)	2.20 (1.49, 3.27)	4.17 (2.84, 6.12)	< 0.001
	Fully adjusted	Reference	1.13 (0.73, 1.74)	1.95 (1.31, 2.91)	3.48 (2.30, 5.19)	< 0.001
Age ≥ 75	Events/N	24/405	69/739	86/791	70/531	
	Rate per 1000 person-years	6.08	10.2	13.08	16.76	
	Unadjusted	Reference	1.71 (1.08, 2.72)	2.27 (1.44, 3.57)	2.97 (1.86, 4.72)	< 0.001
	Fully adjusted	Reference	1.59 (1.00, 2.54)	2.14 [1.35, 3.38]	2.87 (1.78, 4.61)	< 0.001

There was a statistically significant interaction with age and AL quartiles (p-for-interaction < 0.001). Stratified findings according to age are shown in Table 2. Among all 3 age subgroups, the unadjusted incident rate of HF increased in a graded fashion with higher AL quartiles; and the HR increased in a graded fashion with higher AL quartiles in the fully adjusted models. Notably, the unadjusted incident rates of HF within each AL quartile were highest among those aged at least 75 years (compared to those aged < 65 years and those aged 65–74 years); but the fully adjusted HRs for each quartile were highest among those aged < 65 years (compared to those aged 65–74 years and those aged at least 75 years). Supplemental Table 2 shows sequential models for each subgroup, consistently demonstrating mild attenuation with each set of covariates and persistence of an association.

In a fully adjusted model with adjustment for CAD, the HRs for an incident HF event attenuated, but remained statistically significant and increased in a graded fashion similar to the main Cox models: Q2 HR 1.46 95% CI 1.10–1.94; Q3 HR 2.40 95% CI 1.84–3.14; Q4 HR 4.09 95% CI 3.14–5.34 (Supplemental Table 3).

### HF subtypes: HFrEF

Participants experienced 307 HFrEF hospitalization events. The unadjusted incident rate of HFrEF for AL quartile 1 (Q1) was 0.6 per 1000 person-years, for Q2 was 1.23 per 1000 person-years, for Q3 was 2.41 per 1000 person-years, and for Q4 was 4.03 per 1000 person-years. In a fully adjusted model, the HR for an incident HFrEF event increased in a graded fashion with higher AL quartiles: Q2 HR 1.67 95% CI 1.06–2.65; Q3 HR 3.08 95% CI 2.00–4.74; Q4 HR 5.10 95% CI 3.32–7.84 (Table 3). Supplemental Table 4 shows models with sequentially adjustment of covariates, demonstrating that the addition of each set of covariates led to mild attenuation with persistence of an association.

**Table 3:** Association between allostatic load and HF subtypes

		Q1	Q2	Q3	Q4	*P*-Value
		HR (95% CI)	HR (95% CI)	HR (95% CI)	HR (95% CI)	
Incident HFrEF	Rate per 1000 person-years	0.6	1.23	2.41	4.03	
	Unadjusted	Reference	2.06 (1.31, 3.25)	4.07 (2.66, 6.22)	6.86 (4.52, 10.42)	< 0.001
	Fully adjusted	Reference	1.67 (1.06, 2.65)	3.08 (2.00, 4.74)	5.10 (3.32, 7.84)	< 0.001
Incident HFpEF	Rate per 1000 person-years	0.65	1.06	1.87	3.29	
	Unadjusted	Reference	1.66 (1.05, 2.62)	2.96 (1.94, 4.53)	5.31 (3.51, 8.04)	< 0.001
	Fully adjusted	Reference	1.28 (0.81, 2.03)	2.07 (1.34, 3.19)	3.67 (2.39, 5.63)	< 0.001

We repeated this analysis using the classification of HFrEF as EF ≤ 40%, and saw similar results (Supplemental Table 5).

### HF subtypes: HFpEF

Participants experienced 256 HFpEF hospitalization events. The unadjusted incident of HFpEF for Q1 was 0.65 per 1000 person-years, for Q2 was 1.06 per 1000 person-years, for Q3 was 1.87 per 1000 person-years, and for Q4 was 3.29 per 1000 person-years. In a fully adjusted model, the HR for an incident HF event increased in a graded fashion with higher AL quartiles: Q2 HR 1.28 95% CI 0.81–2.03; Q3 HR 2.07 95% CI 1.34–3.19; Q4 HR 3.67 95% CI 2.39–5.63 (Table 2). Supplemental Table 4 shows models with sequentially adjustment of covariates, demonstrating that the addition of each set of covariates led to mild attenuation with persistence of an association.

### Sensitivity analysis

We conducted multiple sensitivity analyses to ensure our findings were robust to analytic decisions. First, we performed an analysis for multiple imputation for the exposure and covariates. Our results were largely similar to our main analyses (Supplemental Table 6). When using a Fine and Gray competing risk model to account for mortality, our results were nearly unchanged (Supplemental Table 7).

## Discussion

Our analysis of REGARDS participants without HF at baseline revealed two important findings. First, we found that higher AL burden was associated with incident HF events, regardless of HF subtype. Second, we found that the associations were strongest among participants younger than 65 years. To the best of our knowledge, this is the first study examining this association, which has important implications on underlying pathophysiology of HF and the development of strategies to prevent HF.

AL has been conceptualized as a physiologic state that represents the cumulative maladaptive burden of stress, including both repeated acute and ongoing chronic stressors [16]. Stressors can be environmental, psychosocial, or economical [8, 16]. Prior work has operationalized AL using physiologic biomarkers, and shown that AL is associated with cancer-related, diabetes-related, and all-cause mortality [9, 10, 25, 26]. To our knowledge, this is the first study to examine and subsequently show the link between AL and incident HF. Our prior work has shown that the burden of social determinants of health is associated with incident HF, a finding that we hypothesized could relate to AL [5]. Findings here now lend credence to this notion. This has important implications—namely, that AL should be considered a risk factor for incident HF and should be considered when developing strategies for HF prevention.

An important challenge of studying AL is that it can be difficult to distinguish AL from traditional risk factors. For this study, we used population-based quartiles to calculate AL score, in an effort to capture mechanisms independent of known risk factors of HF (i.e. hypertension, hyperlipidemia). Toward this end, we found that AL scores based on population-based quartiles (instead of pathologic cutoffs) were associated with incident HF. Even the 2^nd^ quartile of AL (which included levels of heart rate, blood pressure, cholesterol, glucose that were within normal limits) was associated with incident HF (Supplemental Table 1). These elevated but non-pathologic levels indicate that that there are likely mechanisms at play that go beyond traditional risk factors for HF such as hypertension and hyperlipidemia. In addition, we found that the association between AL and incident HF was independent of lifestyle behaviors such as tobacco smoking, alcohol use, physical activity, and diet; suggesting that lifestyle does not explain our findings. Although we did not incorporate data to account for genetic predisposition, prior work has shown that the attributable risk of genetics toward incident HF is minor, suggesting that genetics are unlikely to explain our findings, and further supporting the likelihood that the environment is playing a prominent role [27]. Based on these data, we assert that AL may very well be that critical upstream process driving the observed associations; and that, through its complex interplay between physiology and social factors, [7, 10, 17, 28] AL merits additional attention. Our work thus highlights the need for a paradigm shift where social and environmental risk factors are integrated into risk prediction alongside physiologic factors [29, 30]. For example, structural racism [31] and adverse childhood events [32] have now been recognized as important risk factors for cardiovascular disease. Future work should examine whether the concept of AL can be leveraged toward understanding its effects on the subsequent development of conditions like HF. Such studies could help to advance efforts to operationalize and integrate the effects of the social environment into risk assessments.

The mechanisms by which AL predisposes and possibly leads to incident HF requires further investigation. It is likely that stress and inflammation play an important role, as they are linked closely with high AL burden [7, 8] and are also well-described contributors for the pathogenesis of HF. Whether stress and/or inflammation represent potential therapeutic targets for preventing HF among individuals with high AL is unknown. Given our finding that the association between AL and incident HF remained even after controlling for CAD suggests that the resulting physiologic consequences of AL go beyond epicardial CAD. There is emerging data about factors that drive coronary microvascular dysfunction [33–35], and it is plausible that AL plays an underrecognized role here. Studies that incorporate novel techniques like PET scan [35] could provide insight on this potential link. Our data calls attention to the urgent need to understand the complex pathways by which AL can impact the incidence of disease. Prior conceptual models indicate that AL include primary mediators which are directly released by the activation of the hypothalamic–pituitary–adrenal axis and sympathetic system, and secondary mediators which are downstream effects on multiple biologic systems including cardiovascular, metabolic, and immune/inflammatory [18, 36]. Our study primarily included secondary mediators. Future work should focus on better characterizing primary, secondary, and even tertiary mediators as they relate to AL, and understanding their complex interactions to inform the development of future strategies to mitigate their effects [37].

AL appeared to have the greatest impact on adults younger than 65 years. This pattern mirrors prior work demonstrating that SDOH may have less of influence on outcomes in older adults. In particular, we have previously shown that the association between SDOH burden and incident HF is attenuated at older age, [5] and that higher degrees of SDOH are not associated with either readmission [14] or death [14] at 90 days following a HF hospitalization among older adults aged at least 65 years (Medicare beneficiaries). Possible explanations include improved access to care related to Medicare eligibility at age 65 years, and survival bias whereby individuals who survive to older age may have protective mechanisms in place to guard against the potential negative effects of SDOH [14, 28]. We believe that these likely explain our findings on AL as well. In addition, it is likely that the influence of other factors like AL become less relevant in the context of advancing age, which is the single strongest epidemiologic risk factor for HF [38, 39]. Taken together, our work emphasizes the need to focus on preventative strategies at younger ages to reduce AL burden and mitigate its effects.

There are several strengths in this study. REGARDS is a robust, prospective cohort, with an oversampling of Black participants in order to assess racial disparities. Our cohort included individuals from a varied geographic region and had a relatively long follow up period. Additionally, REGARDS is well-suited to address this question given the availability of granular physiologic and biologic parameters that reflect the organ systems involved in AL and its longitudinal study design. There are also some important limitations to our study. The study is observational in design, thus causation between AL and incident HF cannot be concluded. Variables such as the demographic, socioeconomic, lifestyle, health habits were self-reported, along with hospitalizations, which potentially introduced recall bias into the variables and outcomes We excluded around 9000 participants who had missing data on parameters needed to calculate AL score. This could have led to selection bias and limited generalizability. However, notably, when using multiple imputation for the missing exposure in a sensitivity analysis, we found similar results. Our main outcome of incident HF was defined by HF hospitalization or HF-related deaths, and did not reflect outpatient diagnoses of HF—this also could have impacted observations. Although we controlled for baseline CAD, we did not control for incident CAD over the course of the study. Incident CAD could be an important mediator for our findings, and thus merits additional investigation in future work. Since there is no universal definition of AL, we chose variables available in the REGARDS baseline assessment that mapped to conceptual models for AL. We included variables previously utilized to study the association between AL and cancer-related mortality within REGARDS, and many of the most frequent variables used in prior work to operationalize AL. Since AL is defined as the cumulative burden of adaptation to stress, AL can accumulate and subsequently change over time. While we could only calculate AL at a single time point in our study, longitudinal changes in AL over time could have an important impact on outcomes and should therefore be examined in future studies.

In conclusion, our study showed that AL is associated with incident HF events, regardless of HF subtype. These findings were consistent across all age groups, but we observed the greatest association of AL with incident HF among adults younger than 65 years. These findings identify AL as an important yet overlooked risk factor for incident HF, and as a potential target for preventative strategies particularly in a younger population.

## Supplementary Information

**Additional file 1: Supplemental Table 1.** Median values of each AL parameter, along with IQR by AL quartile. **Supplemental Table 2.** Association between AL and incident HF events in all participants and in the 3 age subgroups with sequential adjustment for selected covariates in four total models: Model 1: baseline demographics; Model 2: Model 1 + geographic region of residence and race; Model 3: Model 2 + socioeconomic factors defined as annual household income, social support, health insurance coverage, and level of education; Model 4: Model 3 + lifestyle behaviors. **Supplemental Table 3.** Association between AL and HF subtypes, in a fully adjusted model with coronary artery disease, as well as multiple imputation of age, geographic region of residence, race, socioeconomic factors, and lifestyle and health habits. **Supplemental Table 4.** Association between AL and incident HF subtype in all participants with sequential adjustment for selected covariates in four total models. **Supplemental Table 5.** Incidence and association between AL and incident HFrEF, defined as EF < 40% in unadjusted, fully-adjusted, and fully-adjusted with coronary artery disease models. **Supplemental Table 6.** Association between AL and HF subtypes, with multiple imputation of age, geographic region of residence, race, socioeconomic factors, and lifestyle and health habits. **Supplemental Table 7.** Multivariable adjusted Cox proportional subdistribution hazard ratiosfor the association between AL and incident HF using the Fine and Gray model for competing risk mortality.

## Data Availability

The datasets generated and/or analyzed during the current study are not publicly available, as the data includes characteristics that may compromise individual patient privacy; but are available from the corresponding author on reasonable request.

## Abbreviations

AL: Allostatic load
HF: Heart failure
SDOH: Social determinants of health
HFrEF: Heart failure with reduced ejection fraction
HFpEF: Heart failure with preserved ejection fraction
REGARDS: Reasons for Geographic and Racial Differences in Stroke
HDL: High density lipoprotein
Q1-Q4: Allostatic load quartiles 1–4
EF: Ejection fraction
CAD: Coronary artery disease
