# Supplementary material for: Allostatic load and incident heart failure in the Reasons for Geographic and Racial Differences in Stroke (REGARDS) study
Source: BMC Cardiovasc Disord. 2023 Jul 4;23:340. doi: 10.1186/s12872-023-03371-z (PMC10318712; doi:10.1186/s12872-023-03371-z)
Supplement: Supplementary file 1 — Additional file 1: Supplemental Table 1. Median values of each AL parameter, along with IQR by AL quartile. Supplemental Table 2. Association between AL and incident HF events in all participants and in the 3 age subgroups with sequential adjustment for selected covariates in four total models: Model 1: baseline demographics; Model 2: Model 1 + geographic region of residence and race; Model 3: Model 2 + socioeconomic factors defined as annual household income, social support, health insurance coverage, and level of education; Model 4: Model 3 + lifestyle behaviors. Supplemental Table 3. Association between AL and HF subtypes, in a fully adjusted model with coronary artery disease, as well as multiple imputation of age, geographic region of residence, race, socioeconomic factors, and lifestyle and health habits. Supplemental Table 4. Association between AL and incident HF subtype in all participants with sequential adjustment for selected covariates in four total models. Supplemental Table 5. Incidence and association between AL and incident HFrEF, defined as EF < 40% in unadjusted, fully-adjusted, and fully-adjusted with coronary artery disease models. Supplemental Table 6. Association between AL and HF subtypes, with multiple imputation of age, geographic region of residence, race, socioeconomic factors, and lifestyle and health habits. Supplemental Table 7. Multivariable adjusted Cox proportional subdistribution hazard ratiosfor the association between AL and incident HF using the Fine and Gray model for competing risk mortality. [file 12872_2023_3371_MOESM1_ESM.docx]

SUPPLEMENTAL MATERIAL

|  |  |  |  |  |  |
| --- | --- | --- | --- | --- | --- |
|  | Q1 | Q2 | Q3 | Q4 | P-value |
| AL Parameter | Median [IQR] | Median [IQR] | Median [IQR] | Median [IQR] |  |
| Heart rate (beats per minute, from ECG) | 61 [55, 67] | 64 [58, 71] | 68 [60, 75] | 71 [65, 79] | <0.001 |
| Systolic blood pressure (mmHg) | 115 [107, 122] | 122 [117, 131] | 129 [120, 139] | 138 [128, 146] | <0.001 |
| Diastolic blood pressure (mmHg) | 71 [65, 77] | 76 [70, 80] | 79 [72, 83] | 82 [78, 88] | <0.001 |
| Total Cholesterol (mg/dL) | 186 [164, 210] | 188 [163, 216] | 191 [166, 219] | 198 [171, 226] | <0.001 |
| HDL Cholesterol (mg/dL) | 59 [49, 71] | 52 [43, 64] | 49 [41, 59] | 44 [37, 53] | <0.001 |
| Albumin (g/dL) | 4.30 [4.10, 4.50] | 4.20 [4.00, 4.40] | 4.1 [3.90, 4.40] | 4.00 [3.80, 4.20] | <0.001 |
| C reactive protein (mg/L) | 0.88 [0.50, 1.7] | 1.70 [0.86, 3.40] | 3.10 [1.40, 6] | 5.20 [2.70, 9.10] | <0.001 |
| Urinary Albumin/Creatinine ratio (mg/g) | 5.10 [3.80, 7.80] | 6.30 [4.40, 11] | 7.9 [5, 16] | 14 [7.10, 38] | <0.001 |
| Cystatin C (mg/L) | 0.83 [0.75, 0.92] | 0.90 [0.80, 1.00] | 0.96 [0.85, 1.10] | 1 [0.91, 1.20] | <0.001 |
| Waist circumference (cm) | 84 [76, 91] | 91 [84, 99] | 97 [89, 107] | 105 [97, 114] | <0.001 |
| Glucose (mg/dL) | 88 [83, 94] | 92 [86, 101] | 97 [90, 109] | 107 [95, 132] | <0.001 |

Supplemental Table 1. Median values of each AL parameter, along with IQR by AL quartile (Q1-Q4).

Supplemental Table 2. Association between AL and incident HF events in all participants and in the 3 age subgroups with sequential adjustment for selected covariates in four total models: Model 1: baseline demographics (age, sex assigned at birth); Model 2: Model 1 + geographic region of residence and race; Model 3: Model 2 + socioeconomic factors defined as annual household income, social support, health insurance coverage, and level of education; Model 4: Model 3 + lifestyle behaviors (tobacco smoking, alcohol use, physical activity and adherence to DASH diet).

|  |  | Q1 | Q2 | Q3 | Q4 | P-Value |
| --- | --- | --- | --- | --- | --- | --- |
|  |  | HR (95% CI) | HR (95% CI) | HR (95% CI) | HR (95% CI) |  |
| All | Crude | Reference | 1.97 (1.49, 2.61) | 3.58 (2.75, 4.66) | 6.25 (4.82, 8.08) | <0.001 |
|  | Model 1 | Reference | 1.62 (1.22, 2.15) | 2.86 (2.20, 3.73) | 5.28 (4.07, 6.83) | <0.001 |
|  | Model 2 | Reference | 1.61 (1.22, 2.14) | 2.85 (2.19, 3.72) | 5.25 (4.04, 6.82) | <0.001 |
|  | Model 3 | Reference | 1.53 (1.16, 2.04) | 2.62 (2.00, 3.41) | 4.68 (3.59, 6.08) | <0.001 |
|  | Model 4 | Reference | 1.49 (1.12, 1.98) | 2.47 (1.89, 3.23) | 4.28 (3.28, 5.59) | <0.001 |
| Age < 65 | Crude | Reference | 2.23 (1.17, 4.25) | 5.99 (3.35, 10.70) | 14.12 (8.09, 24.66) | <0.001 |
|  | Model 1 | Reference | 2.10 (1.10, 4.01) | 5.64 (3.15, 10.10) | 13.33 (7.63, 23.29) | <0.001 |
|  | Model 2 | Reference | 2.02 (1.06, 3.86) | 5.29 (2.95, 9.49) | 12.29 (7.00, 21.58) | <0.001 |
|  | Model 3 | Reference | 1.87 (0.98, 3.57) | 4.56 (2.54, 8.20) | 10.12 (5.74, 17.82) | <0.001 |
|  | Model 4 | Reference | 1.78 (0.93, 3.42) | 4.20 (2.33, 7.59) | 9.16 (5.17, 16.23) | <0.001 |
| Age 65 - 74 | Crude | Reference | 1.18 (0.77, 1.82) | 2.20 (1.49, 3.27) | 4.17 (2.84, 6.12) | <0.001 |
|  | Model 1 | Reference | 1.19 (0.77, 1.83) | 2.20 (1.49, 3.26) | 4.24 (2.89, 6.22) | <0.001 |
|  | Model 2 | Reference | 1.19 (0.77, 1.83) | 2.19 (1.48, 3.26) | 4.20 (2.86, 6.18) | <0.001 |
|  | Model 3 | Reference | 1.16 (0.75, 1.79) | 2.09 (1.40, 3.10) | 3.91 (2.65, 5.79) | <0.001 |
|  | Model 4 | Reference | 1.13 (0.72, 1.74) | 1.95 (1.31, 2.91) | (3.48, 2.34, 5.19) | <0.001 |
| Age > 75 | Crude | Reference | 1.71 (1.08, 2.72) | 2.27 (1.44, 3.57) | 2.97 (1.86, 4.72) | <0.001 |
|  | Model 1 | Reference | 1.67 (1.05, 2.66) | 2.25 (1.43, 3.54) | 2.98 (1.87, 4.75) | <0.001 |
|  | Model 2 | Reference | 1.72 (1.08, 2.74) | 2.37 (1.50, 3.74) | 3.21 (2.01, 5.14) | <0.001 |
|  | Model 3 | Reference | 1.63 (1.02, 2.60) | 2.22 (1.41, 3.51) | 3.01 (1.88, 4.82) | <0.001 |
|  | Model 4 | Reference | 1.59 (1.00, 2.54) | 2.14 (1.35, 3.38) | 2.87 (1.78, 4.61) | <0.001 |

Supplemental Table 3. Association between AL and HF subtypes, in a fully adjusted model with coronary artery disease (CAD), as well as multiple imputation of age, geographic region of residence, race, socioeconomic factors (defined as annual household income, highest level of education achieved, social isolation status, and health insurance coverage), and lifestyle and health habits (defined as smoking status, alcohol use, level of exercise, and adherence to DASH diet)

|  |  | Q1 | Q2 | Q3 | Q4 | P-Value |
| --- | --- | --- | --- | --- | --- | --- |
|  |  | HR (95% CI) | HR (95% CI) | HR (95% CI) | HR (95% CI) |  |
| Incident HF | Fully adjusted + CAD | Reference | 1.46 (1.10, 1.94) | 2.40 (1.84, 3.14) | 4.09 (3.14, 5.34) | <0.001 |
| Incident HFrEF | Fully adjusted + CAD | Reference | 1.63 (1.03, 2.59) | 2.99 (1.94, 4.60) | 4.80 (3.12, 7.39) | <0.001 |
| Incident HFpEF | Fully adjusted + CAD | Reference | 1.24 (0.78, 1.97) | 2.02 (1.31, 3.13) | 3.50 (2.28, 5.39) | <0.001 |

Supplemental Table 4. Association between AL and incident HF subtype in all participants with sequential adjustment for selected covariates in four total models.

|  |  | Q1 | Q2 | Q3 | Q4 | P-Value |
| --- | --- | --- | --- | --- | --- | --- |
|  |  | HR (95% CI) | HR (95% CI) | HR (95% CI) | HR (95% CI) |  |
| Incident HFrEF | Crude | Reference | 2.06 (1.31, 3.25) | 4.07 (2.66, 6.22) | 6.86 (4.52, 10.42) | <0.001 |
|  | Model 1 | Reference | 1.81 (1.14, 2.86) | 3.53 (2.31, 5.41) | 6.25 (4.11, 9.50) | <0.001 |
|  | Model 2 | Reference | 1.81 (1.14, 2.86) | 3.53 (2.31, 5.42) | 6.25 (4.10, 9.54) | <0.001 |
|  | Model 3 | Reference | 1.73 (1.09, 2.74) | 3.29 (2.14, 5.05) | 5.68 (3.71, 8.68) | <0.001 |
|  | Model 4 | Reference | 1.67 (1.06, 2.65) | 3.08 (2.00, 4.74) | 5.10 (3.32, 7.84) | <0.001 |
| Incident HFpEF | Crude | Reference | 1.66 (1.05, 2.62) | 2.96 (1.94, 4.53) | 5.31 (3.51, 8.04) | <0.001 |
|  | Model 1 | Reference | 1.38 (0.87, 2.19) | 2.40 (1.56, 3.67) | 4.48 (2.96, 6.79) | <0.001 |
|  | Model 2 | Reference | 1.39 (0.88, 2.20) | 2.41 (1.57, 3.70) | 4.50 (2.96, 6.86) | <0.001 |
|  | Model 3 | Reference | 1.32 (0.83, 2.08) | 2.20 (1.43, 3.38) | 3.98 (2.61, 6.09) | <0.001 |
|  | Model 4 | Reference | 1.28 (0.81, 2.03) | 2.07 (1.34, 3.19) | 3.67 (2.39, 5.63) | <0.001 |

Supplemental Table 5. Incidence and association between AL and incident HFrEF, defined as EF < 40% in unadjusted, fully-adjusted, and fully-adjusted with coronary artery disease models.

|  |  | Q1 | Q2 | Q3 | Q4 | P-Value |
| --- | --- | --- | --- | --- | --- | --- |
|  |  | HR (95% CI) | HR (95% CI) | HR (95% CI) | HR (95% CI) |  |
| Incident HFrEF | Rate per 1000 person-years | 0.53 | 1.05 | 1.97 | 3.43 |  |
|  | Unadjusted | Reference | 2.00 (1.23, 3.25) | 3.78 (2.40, 5.94) | 6.71 (4.30,10.46) | <0.001 |
|  | Fully adjusted | Reference | 1.63 (1.00, 2.66) | 2.87 (1.81, 4.55) | 5.00 (3.17, 7.91) | <0.001 |
|  | Fully adjusted + CAD | Reference | 1.59 (0.98, 2.60) | 2.79 (1.76, 4.42) | 4.73 (2.99, 7.48) | <0.001 |

Supplemental Table 6. Association between AL and HF subtypes, with multiple imputation of age, geographic region of residence, race, socioeconomic factors (defined as annual household income, highest level of education achieved, social isolation status, and health insurance coverage), and lifestyle and health habits (defined as smoking status, alcohol use, level of exercise, and adherence to DASH diet).

|  |  | Q1 | Q2 | Q3 | Q4 | P-Value |
| --- | --- | --- | --- | --- | --- | --- |
|  |  | HR (95% CI) | HR (95% CI) | HR (95% CI) | HR (95% CI) |  |
| Incident HF | Unadjusted | Reference | 2.01 (1.60, 2.54) | 3.39 (2.73, 4.21) | 6.23 (5.07, 7.65) | <0.001 |
|  | Fully adjusted | Reference | 1.54 (1.22, 1.95) | 2.36 (1.89, 2.94) | 4.28 (3.46, 5.28) | <0.001 |
| Incident HFrEF | Unadjusted | Reference | 2.18 (1.49, 3.18) | 3.74 (2.61, 5.38) | 6.79 (4.82, 9.57) | <0.001 |
|  | Fully adjusted | Reference | 1.78 (1.22, 2.60) | 2.84 (1.97, 4.09) | 5.12 (3.61, 7.27) | <0.001 |
| Incident HFpEF | Unadjusted | Reference | 1.66 (1.14, 2.42) | 2.78 (1.97, 3.93) | 4.93 (3.53, 6.89) | <0.001 |
|  | Fully adjusted | Reference | 1.29 (0.88, 1.88) | 1.98 (1.39, 2.81) | 3.45 (2.44, 4.87) | <0.001 |

Supplemental Table 7. Multivariable adjusted Cox proportional subdistribution hazard ratios (SHR) for the association between AL and incident HF using the Fine and Gray model for competing risk mortality.

|  |  | Q1 | Q2 | Q3 | Q4 | P-Value |
| --- | --- | --- | --- | --- | --- | --- |
|  |  | SHR (95% CI) | SHR (95% CI) | SHR (95% CI) | SHR (95% CI) |  |
| Incident HF | Unadjusted | Reference | 1.88 (1.42, 2.48) | 3.26 (2.50, 4.24) | 5.38 (4.16, 6.96) | <0.001 |
|  | Fully adjusted | Reference | 1.47 (1.11, 1.95) | 2.34 (1.79, 3.07) | 3.83 (2.93, 5.01) | <0.001 |
